# Draft genome sequences of 14 bacterial isolates from the rhizosphere of bioenergy sorghum

**DOI:** 10.1128/mra.00631-26

**Published:** 2026-08-24

**Authors:** Grace Black, Sneha Couvillion

**Affiliations:** 1 Integrated Discovery Sciences Directorate, Pacific Northwest National Laboratory 6865, Sequim, Washington, USA; University of Strathclyde, Glasgow, United Kingdom

**Keywords:** rhizosphere-inhabiting microbes, sorghum, soil, draft genomes, bacterial isolates

## Abstract

We report the draft genome sequences of a collection of 14 bacterial isolates obtained from the rhizosphere soil of bioenergy sorghum (*Sorghum bicolor* [L.] Moench). These isolates represent the genera *Acidovorax*, *Nocardioides*, *Agrobacterium*, *Peribacillus*, *Caulobacter*, *Pseudomonas*, *Rhizobium*, *Sphingomonas*, *Priestia*, *Dyadobacter*, *Roseomonas*, and *Bacillus*.

## ANNOUNCEMENT

Bioenergy sorghum (*Sorghum bicolor* [L.] Moench) is an annual hybrid biomass C4 crop that is drought- and heat-tolerant (1). Plant roots release a variety of compounds that shape rhizosphere microbial communities and influence plant-microbe interactions (2, 3).

Here, we report the draft genome sequences of 14 bacterial isolates obtained from the rhizosphere of bioenergy sorghum. Bacterial strains were isolated from rhizosphere soil collected from individual pot-grown bioenergy sorghum hybrid TX08001 plants grown in soil from Sequim, Washington, USA (48°4′20.3196″ N, 123°3′5.3676″ W). Soil adhering to the roots was collected, and 25 g of soil was suspended in 25 mL of 1× phosphate-buffered saline and filtered through a 50 µm filter, and serial dilutions up to 1:10^6^ were plated separately on R2A agar (4) and soil extract medium (SEM) agar plates. Plates were incubated at 30°C until visible colonies were observed. SEM agar was prepared by suspending 250 g of rhizosphere soil in 500 mL Milli-Q water. The suspension was shaken, autoclaved, allowed to settle overnight at room temperature, and filtered through a 100 µm filter, and 1.5% agar was added before autoclaving again. Colonies with distinct morphologies were purified by repeated streaking on R2A agar plates under the conditions described above. Pure isolates were grown in liquid R2A media (shaking at 100 rpm at 30°C) for glycerol stock preparation and stored at −80°C. For genome sequencing, isolates were revived from glycerol stocks and grown on R2A agar at 30°C for 7 days prior to DNA extraction. DNA was extracted using the Qiagen DNeasy PowerLyzer Microbial Kit (Hilden, Germany), following the manufacturer’s protocol. Whole-genome sequencing was performed by SeqCenter (Pittsburgh, PA, USA). Sequencing libraries were prepared with the tagmentation-based and PCR-based Illumina DNA Prep Kit and custom IDT 10 bp unique dual indices with a target size of 280 bp. Sequencing was performed on an Illumina NovaSeq X Plus sequencer in multiplexed shared-flow-cell runs producing 2 × 151 bp paired-end reads. Demultiplexing, quality control, and adapter trimming were performed with bcl-convert v4.2.4 (Illumina, Inc) (5). Short read assembly was performed with Unicycler (v0.5.0) (6). Raw sequencing reads were deposited in the National Center for Biotechnology Information (NCBI) Sequence Read Archive (SRA) (7), and the draft genome assemblies were deposited in the NCBI GenBank (8) database and annotated using the NCBI Prokaryotic Genome Annotation Pipeline v6.11 (9). Genome completeness and contamination were obtained from NCBI’s CheckM v1.2.5 (10) analyses for the deposited assemblies. For SRI_022 and SRI_024, completeness and contamination were estimated using the CheckM lineage workflow (Galaxy Version 1.2.5+galaxy0) (11) with default parameters and the o__Rhodospirillales (UID3754) and o__Burkholderiales (UID4000) marker sets, respectively. Assembly statistics, genome quality metrics, and accession numbers for both assemblies and raw reads are summarized in Table 1.

**TABLE 1:** Assembly statistics, genome quality metrics, NCBI taxonomic assignments, and accession numbers for 14 bacterial isolates obtained from the rhizosphere of bioenergy sorghum hybrid TX08001^*a*^

Isolate ID	Initial isolation media	Total reads	Genome size (bp)	Number of contigs	Contig N50 (kb)	Genome coverage	GC%	CheckM completeness (%)	CheckM contamination (%)	NCBI taxonomy (family; genus)	SRA accession #	Genome accession #
SRI_002	R2A	11,404,350	5,659,742	20	612.2	151.113×	64.50	99.4	1.32	Comamonadaceae; *Acidovorax*	SRR38228179	JBYCZW000000000
SRI_004	R2A	12,047,876	5,233,661	51	258.7	172.568×	72.00	97.69	3.6	Nocardioidaceae; *Nocardioides*	SRR38228177	JBYCZV000000000
SRI_005	R2A	9,468,142	5,250,863	28	652.7	135.193×	59.00	99.49	0.29	Rhizobiaceae; *Agrobacterium*	SRR38228197	JBYCZU000000000
SRI_006	R2A	10,587,368	5,620,808	63	279.3	141.227×	40.50	99.35	0.32	Bacillaceae; *Peribacillus*	SRR38228200	JBYCZT000000000
SRI_008	R2A	10,357,952	5,582,890	73	168.5	139.091×	69.00	94.74	5.1	Caulobacteraceae; *Caulobacter*	SRR38228175	JBYCZS000000000
SRI_013	R2A	13,383,968	6,178,591	107	338.5	162.311×	63.00	99.68	1.63	Pseudomonadaceae; *Pseudomonas*	SRR38228198	JBYCZR000000000
SRI_014	R2A	11,333,692	4,499,900	27	348.4	188.873×	68.00	87.63	5.87	Caulobacteraceae; *Caulobacter*	SRR38228701	JBYCZQ000000000
SRI_017	SEM	13,982,312	7,132,772	130	309.8	146.883×	61.00	98.51	1.4	Rhizobiaceae; *Rhizobium*	SRR38228691	JBYCZP000000000
SRI_018	SEM	13,056,558	4,143,758	15	2548	236.302×	68.00	98.8	1.62	Sphingomonadaceae; *Sphingomonas*	SRR38228687	JBYCZO000000000
SRI_019	SEM	11,496,688	5,290,098	46	1618	88.0274×	38.00	99.35	0.38	Bacillaceae; *Priestia*	SRR38228269	JBYCZN000000000
SRI_021	SEM	12,430,346	7,687,497	63	767.2	118.728×	49.50	97.42	4.46	Spirosomataceae; *Dyadobacter*	SRR38228196	JBYCZM000000000
SRI-022	SEM	11,954,342	5,714,725	68	184.2	156.884×	69.50	99.5	1.33	Roseomonadaceae; *Roseomonas*	SRR38228705	JBYCZL000000000
SRI_024	SEM	12,654,466	6,842,218	129	196.3	138.633×	69.00	100	0.03	Sphaerotilaceae; genus undefined	SRR38280310	JBYEPP000000000
SRI_025	SEM	11,391,008	3,670,885	23	889.4	232.615×	41.50	99.12	0.29	Bacillaceae; *Bacillus*	SRR38212139	JBYEGB000000000

^*a*^ Family and genus level taxonomic assignments are those assigned by NCBI in the corresponding GenBank records.

## Data Availability

All sequencing data generated in this study have been deposited in NCBI under BioProject accession number PRJNA1455688. Raw sequencing reads have been deposited in the Sequence Read Archive (SRA), and draft genome assemblies have been deposited in GenBank. Individual SRA and genome accession numbers are provided in Table 1. The draft genome assemblies described here represent the first versions deposited in GenBank.
